# Risk factors and prediction of second primary cancer in primary female non-metastatic breast cancer survivors

**DOI:** 10.18632/aging.103939

**Published:** 2020-10-13

**Authors:** Xiwen Qian, Huixun Jia, Yue Zhang, Bingqing Ma, Guoyou Qin, Zhenyu Wu

**Affiliations:** 1 Department of Biostatistics, School of Public Health, Key Laboratory of Public Health Safety and Collaborative Innovation Center of Social Risks Governance in Health, Fudan University, Shanghai, China; 2 Clinical Research Center, Shanghai General Hospital, Shanghai JiaoTong University School of Medicine, Shanghai, China

**Keywords:** breast cancer, second primary cancer, competing risk, nomogram

## Abstract

This study aimed to investigate the risk factors of second primary cancer among female breast cancer (BC) survivors, with emphasis on the prediction of the individual risk conditioned on the patient’s characteristics. We identified 208,474 BC patients diagnosed between 2004 and 2010 from the Surveillance, Epidemiology and End Results (SEER) database. Subdistribution proportional hazard model and competing-risk nomogram were used to explore the risk factors of second primary BC and non-BC, and to predict the 5- and 10-year probabilities of second primary BC. Model performance was evaluated via calibration curves and decision curve analysis. The overall 3-, 5-, and 10-year cumulative incidences for second primary BC were 0.9%, 1.6% and 4.4%, and for second primary non-BC were 2.3%, 3.9%, and 7.8%, respectively. Age over 70 years at diagnosis, black race, tumor size over 2 cm, negative hormone receptor, mixed histology, localized tumor, lumpectomy alone, and surgeries plus radiotherapy were significantly associated with increased risk of second BC. The risk of second non-BC was only related to age, race and tumor size. The proposed risk model as well as its nomogram was clinically beneficial to identify patients at high risk of developing second primary breast cancer.

## INTRODUCTION

Female breast cancer (BC) is the most common cancer in women. In the US, BC showed the highest incidence among female cancers regardless of race or ethnicity, and the mortality was second only to lung and bronchus cancer between 2012 and 2016 [1]. The American Cancer Society reported that the incidence of female BC increased slightly from 2006 to 2015 by 0.4% annually, and more than 3.5 million women had a history of invasive breast cancer by 2016 [2]. By contrast, the mortality of BC has dropped significantly by 40% from 1989 to 2016 [3], and this is attributed to improvements in early detection, diagnosis, and treatments.

With the increasing number of BC survivors, long-term negative outcomes, such as developing a second primary cancer (SPC), have become a new public health concern. Evidence shows that BC survivors have a higher risk for subsequent cancers than the general population [4–7], particularly during the first 10 years after initial diagnosis [4]. Age [8], race [9], hormone receptor status [10], and lifestyle have been reported to be related to the development of SPC among female BC survivors.

This study aimed to investigate the risk factors of developing second primary BC and non-BC among BC survivors, with emphasis on prediction of the individual probabilities of SPCs conditioned on the patient’s characteristics. In most previous studies, the risk of SPCs in BC survivors was either calculated with standard incidence ratios (SIRs) [6, 11–13], or with hazard ratio in traditional survival analysis [10, 13, 14]. The former is a typical univariate analysis, and focused on the overall risk to the general population, while the latter neglects the impact of death, which precludes the occurrence of SPCs in BC survivors, leading to an upward bias. In our study, the multivariate competing-risk model was used, in which death was considered as a competing event. Furthermore, the competing-risk model-based nomogram was provided as the tool for predicting SPC risks in female BC survivors for clinical convenience.

## RESULTS

The characteristics of the study population are listed in Table 1. Of the 208,474 FBC patients identified, 6,242 (3.0%) developed second primary BC, and 12,350 (5.9%) developed second primary non-BC. There were almost 80 sites developing SPCs in addition to breast. The top-10 most commonly diagnosed tumor sites of second non-BC were lung and bronchus, corpus uteri, melanoma of the skin, thyroid, pancreas, ovary, kidney, urinary bladder, NHL-Nodal and cecum (Supplementary Figure 1). The median and maximum follow-up time were 7.4 and 12 years, respectively.

**Table 1:** Baseline characteristics of study population (*N*=208,474).

**Variable**	**Censored**	**Stratified events, No. (%)**			
		**Second BC**	**Second non-BC**	**Death**	***P***
Total	179185 (86.0)	6242 (3.0)	12350 (5.9)	10697 (5.1)	
**Age at diagnosis**					< 0.001
<50	54470 (30.4)	1678 (26.9)	1617 (13.1)	792 (7.4)	
50~	53303 (29.7)	1612 (25.8)	2543 (20.6)	1444 (13.5)	
60~	45497 (25.4)	1532 (24.5)	3696 (29.9)	2940 (27.5)	
70~	25915 (14.5)	1420 (22.7)	4494 (36.4)	5521 (51.6)	
**Race**					< 0.001
White	145755 (81.3)	4884 (78.2)	10433 (84.5)	8773 (82.0)	
Black	17980 (10.0)	865 (13.9)	1142 (9.2)	1399 (13.1)	
Asian/Pacific Islander	15450 (8.6)	493 (7.9)	775 (6.3)	525 (4.9)	
**Laterality**					0.275
Left	90873 (50.7)	3096 (49.6)	6271 (50.8)	5458 (51.0)	
Right	88312 (49.3)	3146 (50.4)	6079 (49.2)	5239 (49.0)	
**Tumor size (cm)**					< 0.001
< 2	104210 (58.2)	3711 (59.5)	7474 (60.5)	5807 (54.3)	
2 ~	74975 (41.8)	2531 (40.5)	4876 (39.5)	4890 (45.7)	
**Nodal status**					< 0.001
Negative	119287 (66.6)	4409 (70.6)	8719 (70.6)	7081 (66.2)	
Positive	59898 (33.4)	1833 (29.4)	3631 (29.4)	3616 (33.8)	
**HR status**					
Negative	35576 (19.9)	1524 (24.4)	2239 (18.1)	1992 (18.6)	
Positive	143609 (80.1)	4718 (75.6)	10111 (81.9)	8705 (81.4)	
**Histology**					< 0.001
Ductal	138225 (77.1)	4699 (75.3)	9284 (75.2)	8017 (74.9)	
Lobular	12097 (6.8)	410 (6.6)	877 (7.1)	802 (7.5)	
Mixed	18884 (10.5)	736 (11.8)	1403 (11.4)	1131 (10.6)	
Other	9979 (5.6)	397 (6.4)	786 (6.4)	747 (7.0)	
**Grade**					< 0.001
I	38483 (21.5)	1352 (21.7)	2969 (24.0)	2410 (22.5)	
II	74945 (41.8)	2562 (41.0)	5311 (43.0)	4723 (44.2)	
III	64083 (35.8)	2271 (36.4)	3970 (32.1)	3464 (32.4)	
IV	1674 (0.9)	57 (0.9)	100 (0.8)	100 (0.9)	
**Tumor stage**					< 0.001
Localized	116250 (64.9)	4307 (69.0)	8506 (68.9)	6840 (63.9)	
Regional	62935 (35.1)	1935 (31.0)	3844 (31.1)	3857 (36.1)	
**Treatment**					< 0.001
Mastectomy alone	53094 (29.6)	1431 (22.9)	3584 (29.0)	4024 (37.6)	
Mastectomy +Radiotherapy	18898 (10.5)	565 (9.1)	1082 (8.8)	918 (8.6)	
Lumpectomy alone	22661 (12.6)	1056 (16.9)	1734 (14.0)	1666 (15.6)	
Lumpectomy +Radiotherapy	84532 (47.2)	3190 (51.1)	5950 (48.2)	4089 (38.2)	

Abbreviation: BC, breast cancer; HR, hormone receptor.

**Figure 1:**
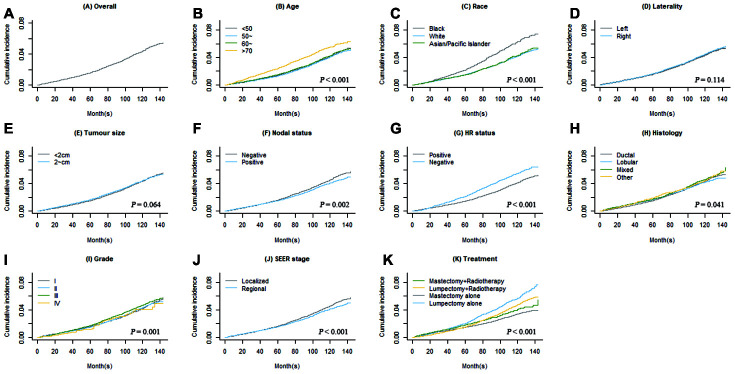
****Overall cumulative incidence function (CIF) curve of second primary breast cancer (BC) (**A**) and CIF curves grouped by each covariate (**B**–**K**).

**Figure 2:**
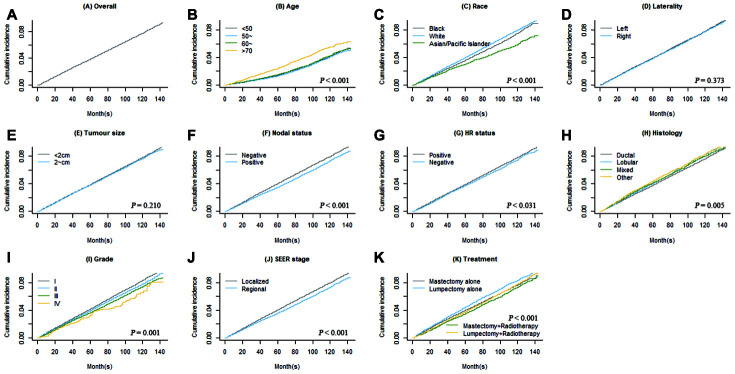
****Overall cumulative incidence function (CIF) curve of second primary non-BC (**A**) and CIF curves grouped by each covariate (**B**–**K**).

CIFs of second primary BC and second primary non-BC are shown in Figures 1 and 2. Regarding death as a competing event, the 3-, 5- and 10-year cumulative incidences of second BC were 0.9%, 1.6% and 4.4%, respectively; the 3-, 5- and 10-year CIFs of second non-BC were 2.3%, 3.9%, and 7.8%, respectively. Female BC patients diagnosed at older age had higher incidence rate of developing SPCs (*P* < 0.001 for both types of SPCs). Race, nodal status, tumor grade, tumor stage, and treatment were also significantly associated with cumulative incidences of both second primary BC and non-BC.

Results for the selected variables in Fine-Gray models are listed in Table 2. Compared with young patients (age < 50), patients aged over 70 years had 47% (sHR=1.47, 95%CI: 1.36 - 1.58) excessive risk of second BC, while the risk of developing second non-BC in patients aged between 50-59, 60-69 and over 70 years were 1.57 times (sHR = 1.59, 95%CI: 1.48 - 1.67), 2.56 times (sHR = 2.59, 95%CI: 2.42 - 2.72) and 4.65 times (sHR = 4.70, 95%CI: 4.39 - 4.93) higher, respectively. Black women had the highest risk (sHR=1.47, 95%CI: 1.36 - 1.58) for second BC compared with white women and Asian/Pacific Islander, and Asian or Pacific Islanders were less likely to develop second non-BC (sHR=0.84, 95%CI: 0.78 - 0.91). Tumor size over 2 cm (sHR = 1.06, 95%CI = 1.01 - 1.12), negative HR (sHR=1.40, 95%CI: 1.32 - 1.49), mixed histology (sHR = 1.16, 95%CI = 1.07 - 1.26), lumpectomy alone (sHR=1.67, 95%CI: 1.55 - 1.81), and surgeries combined with radiotherapy were significantly associated with increased risk of second BC. Tumor size over 2 cm (sHR = 1.09, 95%CI = 1.06 - 1.14) was also related to the risk of second non-BC.

**Table 2:** Factors associated with the risk of second primary breast cancer (BC) and non-BC among BC patients in final predictive models.

	**sHR**	**95% CI**	***P***
***Second primary BC***			
**Age at diagnosis**			
< 50	Ref		
50 ~	0.95	0.89 - 1.02	0.143
60 ~	1.04	0.97 - 1.12	0.267
70 ~	1.47	1.36 - 1.58	< 0.001
**Race**			
White	Ref		
Black	1.47	1.36 - 1.58	< 0.001
Asian/Pacific Islander	1.06	0.98 - 1.17	0.183
**Tumor size**			
<2 cm	Ref		
2~cm	1.06	1.01 - 1.12	0.030
**HR status**			
Positive	Ref		
Negative	1.40	1.32 - 1.49	< 0.001
**Histology**			
Ductal	Ref		
Lobular	1.09	0.98 - 1.21	0.107
Mixed	1.16	1.07 - 1.26	< 0.001
Other	1.03	0.93 - 1.14	0.590
**Tumor stage**			
Localized	Ref		
Regional	0.93	0.87 - 0.99	0.048
**Treatment**			
Mastectomy alone	Ref		
Mastectomy + Radiotherapy	1.27	1.15 - 1.41	< 0.001
Lumpectomy + Radiotherapy	1.37	1.28 - 1.46	< 0.001
Lumpectomy alone	1.67	1.55 - 1.81	< 0.001
***Second Primary non-BC***			
**Age at diagnosis**			
< 50	Ref		
50 ~	1.57	1.48 - 1.67	< 0.001
60 ~	2.56	2.42 - 2.72	< 0.001
70 ~	4.65	4.39 - 4.93	< 0.001
**Race**			
White	Ref		
Black	1.01	0.95 - 1.08	0.716
Asian/Pacific Islander	0.84	0.78 - 0.91	< 0.001
**Tumor size (cm)**			
< 2	Ref		
2 ~	1.09	1.06 - 1.14	< 0.001

Abbreviation: BC, breast cancer; sHR, hazard ratio from subdistribution model; HR, hormone receptor.

**Figure 3:**
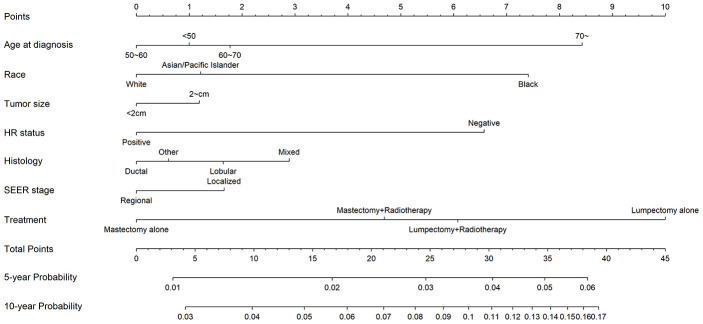
**Competing-risk nomogram for predicting 5- and 10-year risks of second primary breast cancer (BC) in female BC survivors.**

**Figure 4:**
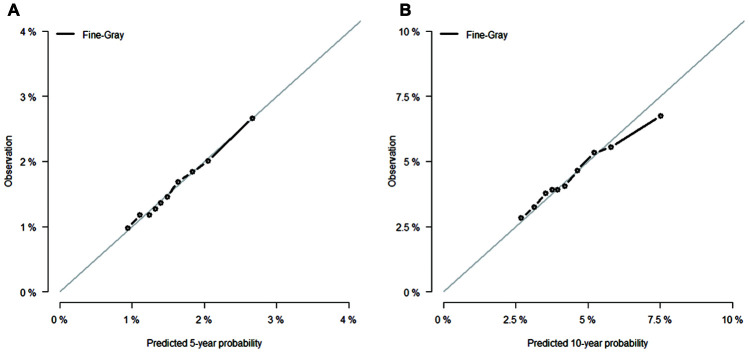
****Calibration curves for (**A**) 5- and (**B**) 10-year predictions from Fine-Gray model. X-axes indicate predicted 5- or 10-year probabilities; Y-axes indicate actual observations.

**Figure 5:**
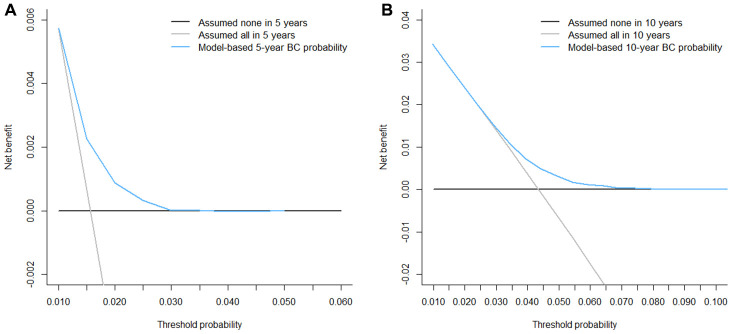
**Decision curve analysis for the risk models for second primary breast cancer (BC).** The decision curves shows that within the threshold probabilities ((**A**) 1.0% - 3.0% for 5-year and (**B**) 2.8% - 7.5% for 10-year prediction of second primary BC, respectively), using the competing-risk model to predict the probability of developing second primary BC can produce more benefit than treating either all or no patient would have second BC.

Competing-risk nomograms for based on the Fine-Gray model were constructed to predict the 5- and 10-year probabilities of second primary BC (Figure 3). Each selected variable is listed separately in the figure, with a corresponding point assigned to a given magnitude of the variable according to the point scale at the top of image. Then for every patient, the total points by summing up points of all variables were matched to the total points scale, which correspond to the 5- and 10-year predictive probabilities at the bottom of image. For example, a 65-year-old black woman was diagnosed with small (< 2 cm), HR-negative, lobular, and localized breast tumor and treated with lumpectomy alone, then she would have 3.8% and 10.4% probabilities of developing a second primary BC after initial diagnosis at 5 years and 10 years, respectively. Calibration curves for 5- and 10-year probabilities of second primary BC indicated good calibration with high correlation between predicted probabilities and observed probability of second BC (Figure 4). The c-index was 0.61 with a moderate discrimination power. DCA was applied to evaluate clinical usefulness of the prediction nomogram. Figure 5 compares the net benefit of the predictive model to those in two hypothetic scenarios: screening all BC survivors and screening none. Within a wide range, the clinical net benefit of the risk model used in 5- and 10-year second BC predictions was larger than that under the assumption of not using the model or screening all patients (Figure 5A and 5B).

## DISCUSSION

To our knowledge, the current study is the largest one to predict the risk of second primary BC and compare the risk factors for second BC and non-BC in female BC survivors. According to our population-based findings, the overall 3-, 5-, and 10-year cumulative incidences for second primary BC were 0.9%, 1.6% and 4.4% respectively, and for second primary non-BC were 2.3%, 3.9%, and 7.8%, respectively. Competing-risk events occur frequently in oncology studies. For example, 5.1% of patients died before developing an SPC in this study. Given that censoring these patients will lead to an overestimated probability of SPCs, the subdistribution competing-risk model was used. For clinical convenience, a competing-risk nomogram was proposed, and the clinical 5- or 10-year risk of developing second BC could be estimated. Identifying subpopulations and individuals with distinct risk of SPCs using the proposed risk model can greatly benefit patients through decision making, because a patient with high risk of SPCs might be asked to consider more subsequent screening, changing adverse living habits or choosing optimal treatment for the current BC to avoid increasing risk from the side effect. Ideally, for those with high risk of developing SPCs, active and regular surveillance could benefit them from early prevention, early detection and early treatment, to improve their quality of life. High-risk BC patients would be identified according to our proposed nomogram, and only for those with high-risk patients, active strategy could be considered. In practice, the implementation of follow-up surveillance strategy not only depends on the risk of subsequent SPC, but also on other factors, such as patient’s financial arrangement, medical insurance and access to healthcare.

Results from the multivariate competing-risk models indicated that age of initial diagnosis and race were significantly related to the risks of both second primary BC and non-BC. Risk differences of age at diagnosis have been extensively examined but remains debatable. Some studies showed that young patients (< 50 years) had greater predisposition to SPCs than the old patients (≥ 50 years) [4, 6, 11, 12, 15]. However, these findings concluded from comparisons between every age group of general population and of the study population without considering other factors simultaneously, thereby SIR cannot comprehensively measure the effect of age. Instead, multivariate Cox proportional hazard regression or its extensions would be more appropriate to identify risk differences based on multiple factors. Our research found that an older age was associated with higher risk of second primary BC and non-BC in BC patients, which is consistent with other multivariate studies [8, 10, 13, 14]. It is well documented that advanced age is among the most important risk factors for many specific cancer types due to the functional decline in immune system, increased susceptibility to carcinogen, reduced DNA repair, and long-term effect of harmful lifestyle. In addition, that older patients tended to develop more SPCs may also affected by less systemic therapy due to their poor physical conditions. Systemic therapy, including chemotherapy, hormonal therapy and immunotherapy, works throughout the entire body for treating or preventing metastasis, and it may also inhibit the development of new tumors before their diagnosis.

Race is also an important demographic predictor for SPC risk. The incidence and risk of second BC was higher among black women than white women and Asian/Pacific Islanders. Other studies also confirmed this racial disparity in the risk of second primary BC [9, 16, 17], which might be explained by the combination of genetic or biologic, clinical and socioeconomic factors. For example, some genetic differences in *BRCA1*, *BRCA2* and *p53* mutations between African Americans and Caucasians in the US have been shown to be associated with different pathological characteristics and tumor prognosis [18]. Moreover, a lower socioeconomic status in any race tends to cause worse tumor pathological characteristics, inappropriate treatments and less healthcare utilization before and after first primary BC [19].

We also observed increased second primary BC risk in BC subtypes classified by clinical tumor characteristics, such as tumor size, HR status, tumor histology and SEER stage. HR-negative BC patients had a higher risk of SPC, which is consistent with previous studies [10, 20, 21]. However, it remains unclear whether the correlation between HR status of the initial BC and risk of second BC reflects an underlying genetic predisposition or exposure to hormone or other risk factors. A review concluded that *BRCA1* mutation carriers were more likely to have triple-negative (ER-, PR- and HER2-negative) BC [22], and these women also had increased risk of developing SPC [23]. In addition, previous studies showed that the distribution of HR status in the female BC patients might be caused by racial or ethnic disparities [24, 25]. Specifically, triple-negative patients were more likely to be non-Hispanic black and Hispanic, while white women had the highest incidence of HR-positive BC subtype. As we discussed previously, black women suffered higher risk of second primary BC than white and Asian or Pacific Islanders; hence, the interaction between HR status and race may partly explain the higher risk of second BC. In summary, it is important to emphasize that in addition to the poor prognosis of HR-negative BC patients, they may also have an elevated risk of developing second primary BC, which would in turn lead to a poor survival. Other studies also examined the association between human epidermal growth factor receptor 2 (HER2) status and the risk of SPC which still remains further research. One previous study reported that those with HER2-overexpressing (ER-/HER2+) and triple-negative BC had elevated risks of developing second primary contralateral breast cancer [21]. A recent study found those with HER2- BC and without adjuvant trastuzumab therapy had higher risk of contralateral breast cancer [26]. While another study claimed that HER2 status did not seem to be a marker of risk for a SPC [27]. For second primary non-BC, it was reported that positive HER2 status increased the risk of digestive system and thyroid tumors [28]. Other pathological features, such as large tumor size, reflected an advanced tumor stage. BC patients diagnosed with advanced tumor might have lived for a longer survival time after tumor cell appeared than patients diagnosed at early stage, so they could have higher risk of recurrence or SPCs. Besides, the misclassification of SPC and metastasis also might happen.

In the current study, the effect of surgical type and radiation treatment were investigated. Lumpectomy plus radiotherapy is one of the common treatment strategies and is likely to be as effective as mastectomy. Although radiotherapy was regarded as an important risk factor for SPCs, it showed no significant effect on non-BC in the final model and less adverse effect than lumpectomy alone on developing second BC. It was reported by other studies that radiotherapy combined with surgery did not increase the risk of second primary BC or non-BC compared to surgery-alone treatment [29], and that radiotherapy after surgery reduced the long-term risk of recurrence [30]. What is more, the progress in technology can provide better radiation quality and dose conformality to target, hence better preserve the adjacent normal tissue and reduce the risk of radiotherapy-related SPCs like lung cancer and leukemia. The decreasing adverse effect of radiotherapy has been reported in serval previous studies [31, 32]. Patients treated with lumpectomy alone had the highest risk of second BC, which may imply the drawback of this surgery type that BC would come back if tumor cells were not removed completely.

The current study had strengths with respect to its methodology and model ability of identifying high-risk SPC patients. Limitations mainly came from the absence of some important variables in the SEER database, such as detailed treatment information including chemotherapy, radicality of breast-conserving surgery and so on, and factors that influence cancer incidence including smoking status, sociodemographic status and genetic factors. A moderate C-index may also result from the absence of detailed information. Besides, the quality of collected data may differ among registry sites over time. Future work will focus on external validation with other populations and perfecting our proposed model by including more covariates.

## CONCLUSIONS

Important risk factors for second primary BC after initial primary BC diagnosis included age at diagnosis, black race, large tumor size, negative HR status, mixed tumor histology, localized tumor and receiving lumpectomy and radiotherapy. While the risk of second primary non-BC was less relevant to initial breast tumor characteristics and treatment. The proposed nomogram was clinically beneficial to identify patients at high risk of developing SPCs, so they may benefit from further efforts targeted at improving access to better healthcare and SPC prevention.

## MATERIALS AND METHODS

### Data source and study population

Data were collected from the Surveillance, Epidemiology, and End Results (SEER) Program (2018 submission). The SEER program collects information on demographics, tumor characteristics, and treatments of patients from various regions in the US since 1973 [33]. Female BC patients were included in the study if they were (1) diagnosed as primary BC, (2) aged between 20 and 80 years, (3) diagnosed before 2011 (to ensure a minimum of 5-year follow-up after initial diagnosis), and (4) diagnosed after 2003 because variables like estrogen receptor (ER) and progesterone receptor (PR) were recorded since 2004. Patients were subsequently excluded if (1) information was obtained from death certificate or autopsy, (2) demographic or clinical information was missing or unknown, (3) treatment information were not recorded or unspecific, (4) distant metastases occurred, and (5) diagnosed with bilateral tumors. A total of 208,474 female non-metastatic BC patients diagnosed between 2004 and 2010 and who were followed up over 5 years were identified as the final study population.

### Definition of SPC

SPC was defined according to the SEER guidelines, which takes into account the tumor site, tumor behavior (in situ or invasive), histology, date of diagnosis, and laterality of paired organs [34]. The SEER rules mainly comprise the followings:

1. A new lesion with same histologic type as the previous tumor in the same site and occurring synchronously (within 2 months) is to be regarded as a single tumor, not a new primary one.
2. A new lesion with different histologic type as the previous tumor in the same site and occurring synchronously (within 2 months) is to be regarded as a new primary tumor.
3. A new lesion in the same site occurring metachronously (2 months or more after the primary diagnosis) is counted as a new primary tumor regardless of histologic type, unless it is confirmed as a metastatic lesion.
4. A new lesion in different site with either the same or different histologic type is to be considered as a new primary tumor regardless of time, unless it is stated to be a metastatic lesion.
5. For paired organs, synchronous bilateral tumor with only one histologic type is considered as a single primary tumor; bilateral tumor with two different histologic types is considered two primaries unless stated to the contrary.

### Outcome and covariates

The primary outcome of the current study was the occurrence of SPC in female BC patients, and SPCs were further classified as second primary BC and second primary non-BC. Death as the result of any cause before SPC was defined as a competing event. Demographic covariates included age at initial diagnosis of BC and race (White, Black and Asian/Pacific Islander). We categorize age into <50, 50~, 60~, and 70~, because 1) female breast cancer patients after menopause are at higher risk of developing a new malignancy [14], 2) menopause is reported starts from the age of 50 in the United States [35], 3) 10-year-old age groups are commonly used in related studies [14, 28, 36]. Tumor characteristic covariates referred to laterality, tumor size, nodal status, hormone receptor (HR) status (Positive: either ER or PR was positive; Negative: both ER and PR were negative), histology (Ductal, Lobular, Mixed and Other), grade (I: well-differentiated, II: moderately-differentiated, III: poorly-differentiated and IV: undifferentiated), SEER stage (Localized and Regional). In this study population, radiotherapy was given to 78.1% of patients treated with lumpectomy, and 72.9% of patients treated with mastectomy were not given radiotherapy. Therefore, treatment was categorized into four types: mastectomy alone, mastectomy plus radiotherapy, lumpectomy alone and lumpectomy plus radiotherapy.

### Statistical analyses

Possible differences of patients’ characteristics between event groups were examined by Chi-square test. The overall cumulative incidence function (CIF) of developing an SPC as well as CIFs grouped by each covariate was calculated. We plotted the CIF curves and performed the Gray’s test to identify differences between groups.

### Competing-risk modelling and nomogram

The Fine-Gray subdistribution proportional hazard model [37], an extension of conventional Cox proportional hazard model, is commonly used in the presence of competing risk and is suggested to be appropriate for quantifying the effect of covariates on the incidence of the outcome and predicting incidence over time [38, 39]. Therefore, in this study, the subdistribution proportional hazard model was applied to explore risk factors and to predict the unbiased risk probability of developing an SPC. Variable selection was determined via stepwise backward elimination method with Akaike information criteria (AIC). Subdistribution hazard ratio (sHR) was used to assess the effect of each covariate on the risk of SPCs. A competing-risk nomogram was subsequently built to predict the 5- and 10-year probabilities of developing SPCs. Each covariate was assigned with a score to indicate the variable importance, and by summing up all scores of selected variables, the predictive probabilities of developing an SPC for a certain BC patient can be obtained.

### Model performance and evaluation

For validation, we used calibration with a bootstrap cross-validation method to evaluate the model performance. The predictive model was trained on 200 bootstrap samples drawn with replacement of the same size as the original data. The model was assessed in the observations that are not in the bootstrap sample. The concordance index (C-index) was calculated to measure the ability of the model to distinguish outcomes. Furthermore, decision curve analysis (DCA) [40], which is a novel method of evaluating diagnostic and prognostic prediction models, was applied to assess the clinical usefulness and magnitude of benefit of the proposed competing-risk model. A model is clinically valuable in a certain range of threshold probabilities, if it produces a larger net benefit through identifying patients with high risk of SPC than assuming all or none of the patients will develop an SPC.

All the statistical analyses and graph plotting were performed in R 3.5.1 (http://www.r-project.org).

## Supplementary Material

Supplementary Figures
