# Supplementary material for: Risk factors and prediction of second primary cancer in primary female non-metastatic breast cancer survivors
Source: Aging (Albany NY). 2020 Oct 13;12(19):19628–40. doi: 10.18632/aging.103939 (PMC7732282; doi:10.18632/aging.103939)
Supplement: Supplementary Figures [file aging-12-103939-s001..pdf]

SUPPLEMENTARY FIGURE

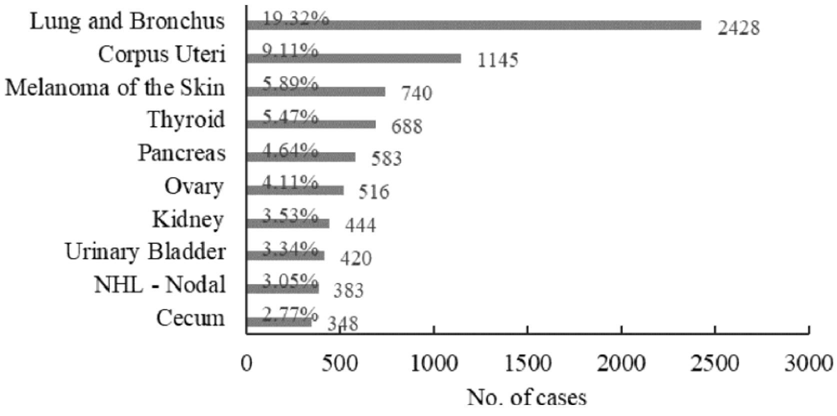

Supplementary Figure 1. The top-10 most commonly diagnosed sites of second primary non-BC.
